# P-326. Barriers to Oral HIV Pre-Exposure Prophylaxis (PrEP) Perceived by Those Receiving an Initial Prescription: US Survey Analysis

**DOI:** 10.1093/ofid/ofaf695.545

**Published:** 2026-01-11

**Authors:** Patrick S Sullivan, JeanPierre Coaquira Castro, Krisha Patel, Alice Hsiao, Iyue Sung, Jessica Citronberg, Michael Bogart, Woodie Zachry

**Affiliations:** Emory University Rollins School of Public Health, Atlanta, GA, USA., Atlanta, GA; Gilead Sciences, Inc., Foster City, CA, United States, Foster City, California; Walgreens Co., Deerfield, IL, USA., Deerfield, Illinois; Gilead Sciences, Inc., Foster City, CA, United States, Foster City, California; Walgreens Co., Deerfield, IL, USA., Deerfield, Illinois; Walgreens Co., Deerfield, IL, USA., Deerfield, Illinois; Gilead Sciences, Inc., Foster City, CA, United States, Foster City, California; Gilead Sciences Inc, Foster City, California

## Abstract

**Background:**

Understanding barriers to PrEP initiation is critical to maximizing access. In a prior pilot study, respondents picking up an initial oral PrEP prescription reported cost and coverage issues. The current study utilizes a larger sample size to more robustly assess reported pharmacy-level barriers to initial dispensation.

**Table 1.**
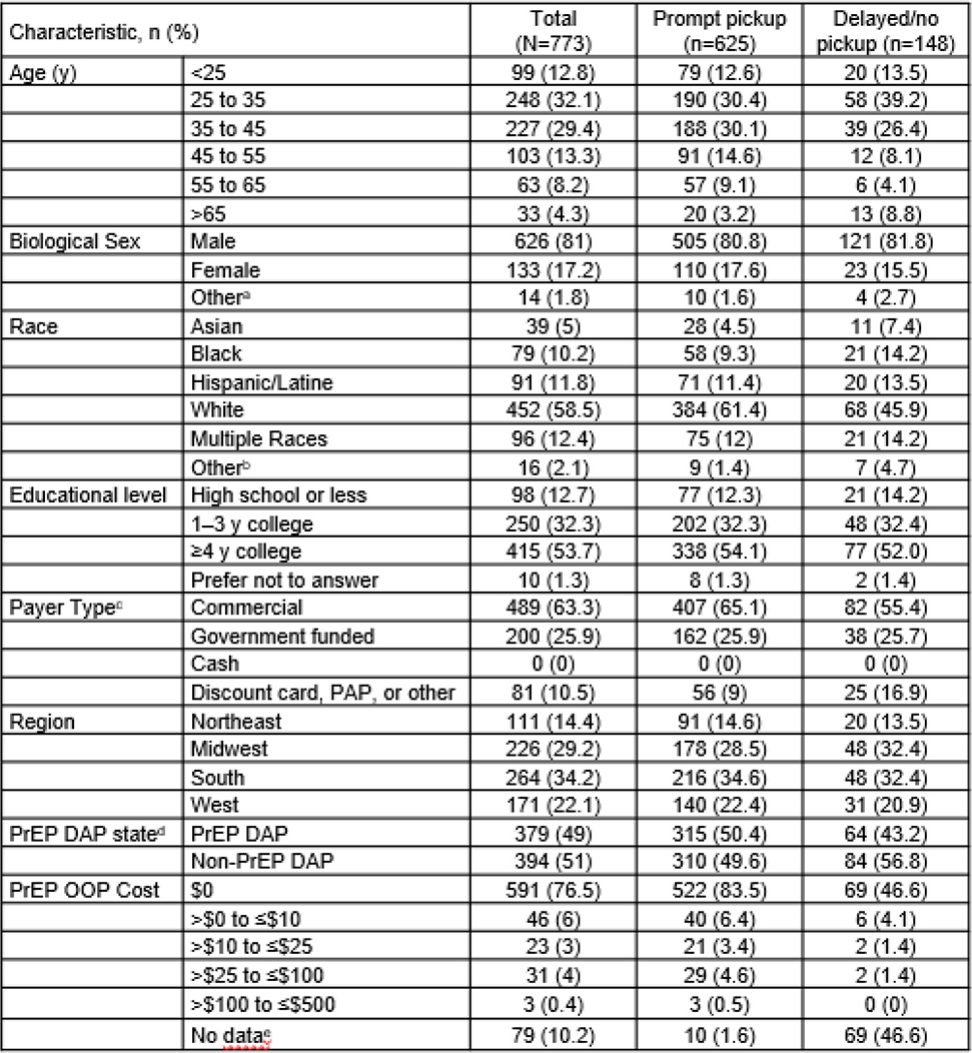
Characteristics of Survey Respondents by PrEP Dispensation Group, Walgreens Pharmacy Clients, United States, 2024 a.Includes none of the above and prefer not to answer. b.Includes American Indian or Alaska Native, Middle Easter or North African, and prefer not to answer. c.Government funded includes Medicare Part D, Medicare Part B, managed Medicaid, and state Medicaid. d.Includes California, Colorado, DC, Florida, Illinois, Indiana, Iowa, Massachusetts, New Mexico, New York, Ohio, Oklahoma, Virginia, and Washington State. e.Cost data unavailable due to no pickup within 30 days or otherwise missing data. DAP, drug assistance program; OOP, out-of-pocket; PAP, patient assistance program; PrEP, pre-exposure prophylaxis; y, years.

**Table 2.**
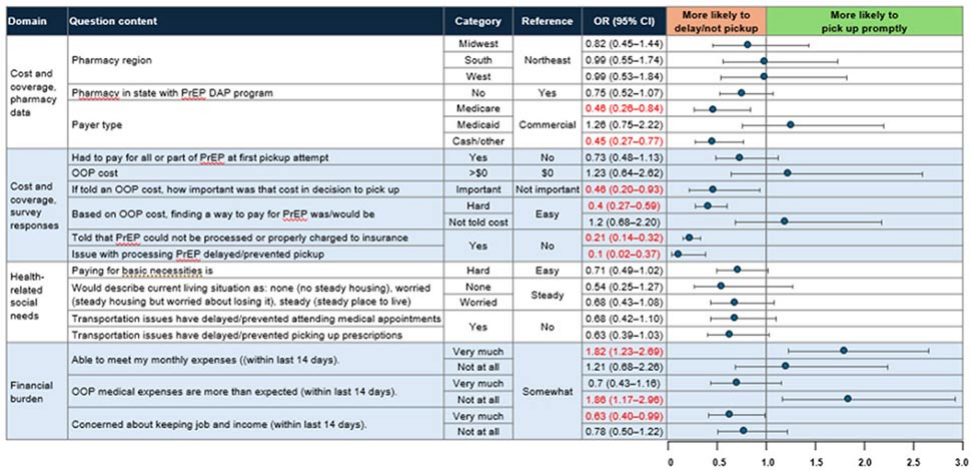
Unadjusted Odds Ratios For PrEP Dispensation Within 14 Days for Variables Related to Cost and Coverage, Walgreens Pharmacy Clients, United States, 2024 Statistical significance indicated by red text. CI, confidence interval; DAP, drug assistance program; OOP, out-of-pocket cost; OR, odds ratio; PrEP, pre-exposure prophylaxis.

**Methods:**

Adults with a recent (≤7 months prior) initial PrEP prescription and no record of HIV medications in the Walgreen’s Pharmacy database were invited to complete a 35 question survey. The survey was deployed electronically (Mar to Dec 2024) to eligible and consenting participants. Topics included attitudes toward PrEP, out-of-pocket (OOP) costs, and prescription fill rejection reasons. Responses were compared between individuals who picked up their first PrEP prescription within 14 days (prompt pickup [PP]) and individuals who did not pick up within 14 days (delayed/no pickup [D/NP]) using t-tests and Chi-square/Fisher’s exact tests (significance threshold p≤0.05). Associations between predictor variables and pickup status were evaluated with univariable (ULR) and multivariable logistic regression (MLR) analysis.

**Table 3.**
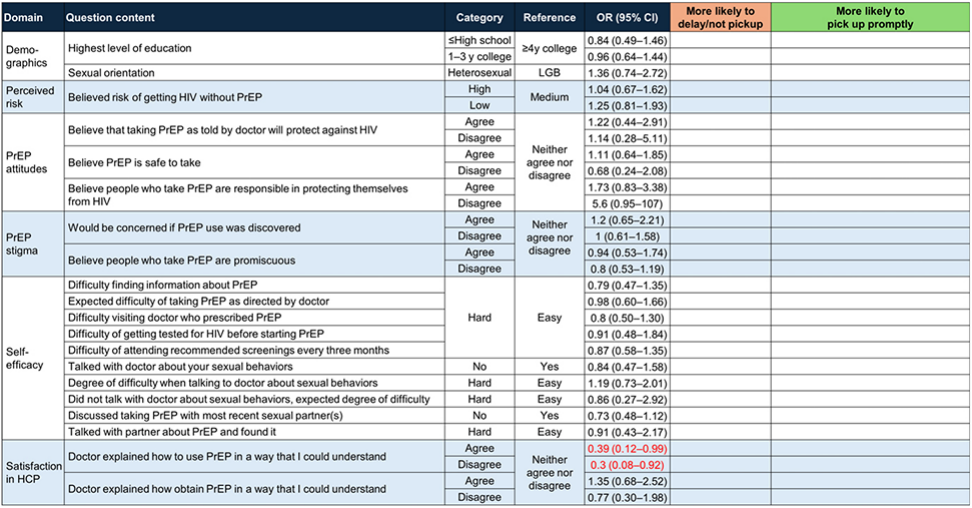
Unadjusted Odds Ratios For PrEP Dispensation Within 14 Days for Variables Related to PrEP Attitudes, Stigma, and Self-Efficacy, Walgreens Pharmacy Clients, United States, 2024 Statistical significance indicated by red text. CI, confidence interval; HCP, healthcare provider; LGB, lesbian, gay, or bisexual; OR, odds ratio; PrEP, pre-exposure prophylaxis; y, years.

**Results:**

Of 39,596 invited individuals, 773 responded; 80.9% (n=625) had PP and 19.1% (n=148) had D/NP. Respondent characteristics were similar between groups, with the exception of OOP costs (Table 1). Among D/NP respondents, 46.6% paid $0 for PrEP and 46.6% had no cost data, primarily due to no pickup, as compared with 83.5% and 1.6%, respectively, of PP respondents. In the ULR analysis, cost and coverage considerations and financial burden were significantly associated with pickup group, in the predicted directions; non-significant associations were observed with health-related social factors (Table 2). MLR results (not shown) were consistent with ULR results. No strong associations were found between pickup group and other assessed factors, including PrEP attitudes, stigma, and self-efficacy (Table 3).

**Conclusion:**

Cost concerns and insurance coverage issues were significantly more prevalent among individuals with D/NP versus PP of their first PrEP prescription. To further encourage PrEP initiation, efforts should target increasing prescription affordability and streamlining medical authorization processes.

**Disclosures:**

Patrick S. Sullivan, DVM, PhD, Gilead Sciences Inc.: Grant/Research Support|Gilead Sciences Inc.: Speaker fees|Merck: Grant/Research Support|ViiV Healthcare: Grant/Research Support JeanPierre Coaquira Castro, MPH, Gilead Sciences, Inc.: employee and shareholder Krisha Patel, BS, MPH, Walgreens Real World Evidence-Clinical Trials: Employee Alice Hsiao, PharmD, Gilead Sciences, Inc.: Employee and shareholder Iyue Sung, PhD, Walgreens Real World Evidence-Clinical Trials.: Employee Jessica Citronberg, PhD, Walgreens Real World Evidence-Clinical Trials: Employee Michael Bogart, n/a, Gilead Sciences, Inc.: Employee|Gilead Sciences, Inc.: Stocks/Bonds (Public Company) Woodie Zachry, RPh, PhD, Gilead Sciences, Inc.: Employee and shareholder

